# Click‐Chemistry (CuAAC) Trimerization of an α_v_β_6_ Integrin Targeting Ga‐68‐Peptide: Enhanced Contrast for in‐Vivo PET Imaging of Human Lung Adenocarcinoma Xenografts

**DOI:** 10.1002/cbic.202000200

**Published:** 2020-06-09

**Authors:** Neil Gerard Quigley, Stefano Tomassi, Francesco Saverio di Leva, Salvatore Di Maro, Frauke Richter, Katja Steiger, Susanne Kossatz, Luciana Marinelli, Johannes Notni

**Affiliations:** ^1^ Institute of Pathology Technische Universität München Trogerstrasse 18 81675 München Germany; ^2^ Dipartimento di Farmacia Università degli Studi di Napoli Federico II Via D. Montesano 49 80131 Napoli Italy; ^3^ Klinik für Nuklearmedizin and TranslaTUM Central Institute for Translational Cancer Research Technische Universität München Ismaninger Str. 22 81675 München Germany; ^4^ Dipartimento di Scienze e Tecnologie Ambientali Biologiche e Farmaceutiche Università degli Studi della Campania “Luigi Vanvitelli” Via A. Vivaldi 43 81100 Caserta Italy

**Keywords:** CuAAC, integrins, positron emission tomography, radionuclides, transforming growth factor beta

## Abstract

α_v_β_6_ Integrin is an epithelial transmembrane protein that recognizes latency‐associated peptide (LAP) and primarily activates transforming growth factor beta (TGF‐β). It is overexpressed in carcinomas (most notably, pancreatic) and other conditions associated with α_v_β_6_ integrin‐dependent TGF‐β dysregulation, such as fibrosis. We have designed a trimeric Ga‐68‐labeled TRAP conjugate of the α_v_β_6_‐specific cyclic pentapeptide SDM17 (cyclo[RGD‐Chg‐E]‐CONH_2_) to enhance α_v_β_6_ integrin affinity as well as target‐specific in‐vivo uptake. Ga‐68‐TRAP(SDM17)_3_ showed a 28‐fold higher α_v_β_6_ affinity than the corresponding monomer Ga‐68‐NOTA‐SDM17 (IC_50_ of 0.26 vs. 7.4 nM, respectively), a 13‐fold higher IC_50_‐based selectivity over the related integrin α_v_β_8_ (factors of 662 vs. 49), and a threefold higher tumor uptake (2.1 vs. 0.66 %ID/g) in biodistribution experiments with H2009 tumor‐bearing SCID mice. The remarkably high tumor/organ ratios (tumor‐to‐blood 11.2; ‐to‐liver 8.7; ‐to‐pancreas 29.7) enabled high‐contrast tumor delineation in PET images. We conclude that Ga‐68‐TRAP(SDM17)_3_ holds promise for improved clinical PET diagnostics of carcinomas and fibrosis.

## Graphical abstract

**See three with glee**: Tethering three rather than a single peptidic α_v_β_6_ integrin ligand to a radionuclide lets tumor tissue light up brightly in tomographic images, potentially enabling a more sensitive diagnosis of various cancers.

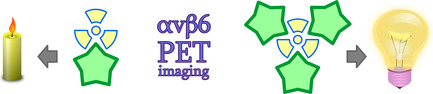

## Introduction

Integrins are a family of 24 heterodimeric transmembrane receptors, each comprising one out of 18 α‐ and one out of eight β‐subunits, which are expressed by almost all animal cells and fulfill a wide variety of biological purposes and functions. Integrins are primarily adhesion receptors that facilitate the binding of cells to extracellular matrix (ECM) proteins, but they are also involved into a variety of signaling processes. A puzzling complexity is observed here because fundamentally different functions are sometimes promoted by binding to the same peptide sequences. For example, α_v_β_6_ integrin shares its ability to recognize the arginine‐glycine‐aspartate (RGD) peptide sequence contained in many ECM proteins and clotting factors with seven other integrins^1^ (such as the popular and well‐characterized vitronectin receptor α_v_β_3_ integrin), but its key function is a fundamentally different one, namely, activation of transforming growth factors β1 and −3 (herein further abbreviated as TGF‐β).^2^

TGF‐betas are a pleiotropic family of phylogenetically old cytokines, a class of signaling proteins that are produced by almost any mammalian cell type.^3^ They are secreted into the extracellular space, albeit not in a freely diffusible form capable of binding to the respective receptors, but as complexes with a temporary inhibitor called latency‐associated peptide (LAP). To exert its biological signaling functions, TGF‐β must be released from this aggregate. This occurs predominantly by binding of the extracellular domain of α_v_β_6_ integrin to a RGD motif in LAP. Within the cell, the β_6_ subunit is connected to the actin filaments of the cytoskeleton and basically acts as a rope to exert a pulling force on LAP, by which the latter is deformed and loses its ability to bind TGF‐β.^4^ As a result, TGF‐β is released and influences gene expression and protein synthesis of adjacent cells.^5^

α_v_β_6_ Integrin expression and ‐signaling is thus an integral part of the TGF‐β‐mediated cell communication mechanism controlling tissue development and homeostasis, whose dysregulation is related to a wide variety of diseases.^3^ Overexpression of α_v_β_6_ integrin has particularly interesting implications in terms of cancer.^6^ For example, TGF‐β acts as a growth suppressor by regulating the transcription of certain growth‐promoting genes. Tumor cells can however acquire a reduced TGF‐β sensitivity as a result of downstream mutations in the signaling pathway,^7^ meaning that their growth is no more inhibited by increased TGF‐β concentrations.^8^ Such tumor cells can benefit from a high TGF‐β concentration in their surrounding because it limits the growth of normal cells and facilitates the tumor to infiltrate healthy tissue. Consequently, a high α_v_β_6_ integrin expression is frequently observed on carcinoma cells,^6^ particularly on pancreatic carcinoma^9^ as well as its metastases and precursor lesions (PanIN).^10^ Likewise, α_v_β_6_ integrin expression is connected to other carcinogenic processes associated with elevated TGF‐β levels, such as epithelial mesenchymal transition (EMT) and metastasis, deposition of ECM proteins and activation of fibroblasts, or the suppression of T‐cell‐mediated immunosurveillance of tumor cells and resistance to immune checkpoint inhibitors.^3^ Hence, α_v_β_6_ integrin represents a promising biomarker for the invasive potential and malignancy of carcinomas. This is however only part of a bigger picture, as dysregulation of the TGF‐β pathway is involved into many other pathogenic processes, for example, inflammation and fibrosis of the lung.^11^

**Figure 1:**
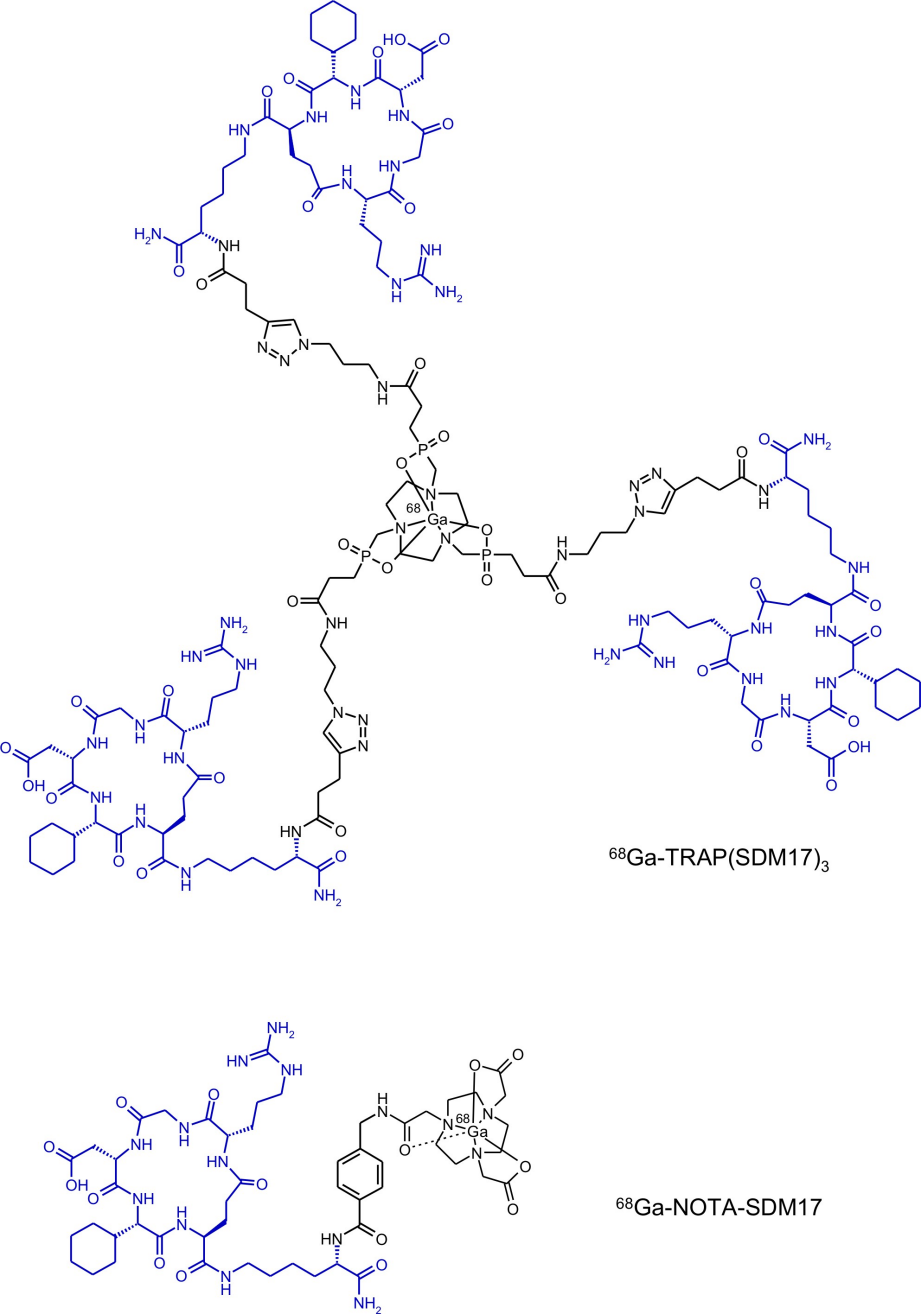
Structures of ^68^Ga‐TRAP(SDM17)_3_ and ^68^Ga‐NOTA‐SDM17.^12^ The SDM17 cyclopeptide unit and the primary Lys linker contained in both conjugates is highlighted in blue.

Targeting α_v_β_6_ integrin might therefore possess a high value for fundamental research and clinical reasoning. In particular, the analysis of spatiotemporal expression patterns in living subjects by means of quantitative noninvasive 3D imaging might guide the way towards a deeper understanding of the activation of TGF‐β in the course of various diseases, or enable a better prognosis for carcinoma patients. To this end, we earlier reported SDM17 (cyclo‐[RGD‐Chg‐E]‐CONH_2_), a small, cyclic pentapeptide as a selective ligand for this target.^12^ Initially, we successfully used a radiolabeled conjugate of this peptide, ^68^Ga‐NOTA‐SDM17 (Figure 1), to demonstrate the feasibility of in‐vivo imaging of α_v_β_6_ integrin expressing subcutaneous xenografts in mice by means of positron emission tomography (PET).^12^ However, this compound exhibited an unsatisfyingly low affinity, resulting in a comparably low uptake in the tumor. Based on the observation that the affinity and the tumor uptake of ligands for RGD‐binding integrins can be substantially increased by multimerization,^13^ we hypothesized that a trimeric conjugate of SDM17 should exhibit an improved uptake and imaging performance. Hence, we investigated the trimer ^68^Ga‐TRAP(SDM17)_3_ (Figure 1) in terms of its suitability for in‐vivo PET imaging of α_v_β_6_ integrin.

## Results and Discussion

### Synthesis

In the monomer NOTA‐SDM17,^12^ a single SDM17 cyclopeptide unit is linked via *p*‐aminobenzoic amide to the 1,4,7‐triazacyclononane‐1,4,7‐triacetic acid (NOTA) chelator via amide bonds (Figure 1). It is worth noticing that the actual chelating unit is not NOTA but NOTA‐monoamide, since the linker connects as an amide to one of the *N* pendant arms of NOTA.

**Scheme 1:**
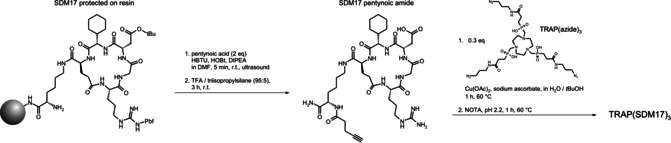
Synthesis of SDM17 pentynoic amide and TRAP(SDM17)_3_, starting from protected resin‐bound SDM17.^12^

In contrast, the trimer was elaborated on the basis of the triazacyclononane‐triphosphinate (TRAP) chelator scaffold^14^, ^15^ employing click chemistry (CuAAC).^16^ For this purpose, SDM17 was decorated on‐resin with 4‐pentynoic acid, employing a ultrasound‐enhanced coupling protocol which enables reaction times as short as 5 min.^17^ Following simultaneous cleavage off the resin/deprotection and purification, 3.3 equivalents of the intermediate, SDM17 pentynoic amide, were reacted with the threefold azide‐decorated TRAP building block TRAP(azide)_3_. The CuAAC trimerization was carried out applying a robust and convenient one‐pot protocol which includes the final removal of any TRAP‐bound copper by transchelation with NOTA (Scheme 1).^18^

### Radiochemistry

A radiolabel for PET imaging was introduced by complexation of the positron emitter gallium‐68 (*t*_1/2_=68 min),^19^ which is conveniently available from ^68^Ge/^68^Ga radionuclide generators (small, commercially available benchtop devices acting as long‐lived regenerative sources for ^68^Ga^III^ in dilute HCl)^20^ and which is particularly suited for straightforward and cost‐efficient development of PET radiopharmaceuticals.^21^ The TRAP structural motif is characterized by particularly robust and efficient ^68^Ga labeling,^22^, ^23^ because its rapid Ga^III^ incorporation reaction is comparably insensitive towards high concentrations of frequently occurring metal ion contaminants in generator eluates and ^68^Ga labeling solutions, such as Fe^III^,^24^ Zn^II^, or Cu^II^.^25^ Accordingly, ^68^Ga labeling of 5 nmol of TRAP(SDM17)_3_ in a total reaction volume of approximately 1.5 mL for 2–3 min at pH 2 and 95 °C reliably afforded ^68^Ga‐TRAP(SDM17)_3_ in >96 % radiochemical yield with a purity of >99.5 % according to radio‐TLC (Figure S3 in the Supporting Information).

Although being somewhat less efficient than TRAP,^15^, ^22^ NOTA is also known as an excellent ^68^Ga^III^ chelating system. However, we found that a relatively high concentration of NOTA‐SDM17 (10 nmol in 1.5 mL, pH 3, 95 °C, 5 min) was necessary to obtain acceptable radiolabeling yields (averagely 76 %, range 70–81 %, *n*=4). Actually, this is worse than what would be expected for an average DOTA peptide (DOTA=1,4,7,10‐tetraazacyclododecane‐1,4,7,10‐tetraacetic acid), as approximate amounts of only 5, 1, or 0.2 nmol of DOTA‐, NODAGA‐, or TRAP conjugates, respectively, are required under otherwise similar conditions to achieve a comparable percentage of ^68^Ga incorporation.^22^ Moreover, we found that the resulting radiopharmaceutical still contained a small fraction of radiochemical impurity even after purification (according to radio‐TLC, ca. 2.5–3 % of noncomplexed ^68^Ga^III^; Figures S4 and S5); this had a noticeable impact on biodistribution (see below).

Apparently, the NOTA structural motif only possesses a high ^68^Ga^III^ chelation efficiency if none of its *N*‐acetic acid substituents is used for conjugation, that is, all three carboxylates are available for coordination. This might appear somewhat counterintuitive, since sacrificing one carboxylate for conjugation does not substantially impair the functionality of its larger congener DOTA.^26^ This, however, works only because the resulting DOTA‐monoamide motif retains three negative formal charges to counterbalance the +3 charge of the metal ion, keeping it capable of rapidly forming kinetically inert ^68^Ga^III^ complexes.^27^ Our observations suggest that for an optimal radiolabeling performance of NOTA in bioconjugates, a separate pendant arm with an additional functional group dedicated to conjugation is indispensable, preferably attached to one of its backbone or *N*‐acetic acid methylene groups, such as in its bifunctional derivative NODAGA.^28^ Conjugation of NOTA by amide bonding on one of the acetic acid N‐substituents, essentially resulting in a NOTA‐monoamide chelator structure, yields constructs with poor ^68^Ga labeling properties and is thus not recommendable, despite such compounds have even been translated into humans.^29^

### In‐vitro characterization

Consistent with previous experience,^30^ ^68^Ga‐TRAP(SDM17)_3_ possesses an approximately 28‐fold higher α_v_β_6_ integrin affinity (IC_50_=0.26 nM) than the monomer ^68^Ga‐NOTA‐SDM17 (7.4 nM) as determined by ELISA on immobilized integrins^31^ (Table 1). Interestingly, the trimer also exhibits a 13‐fold higher selectivity over the functionally related integrin subtype α_v_β_8_;^32^, ^33^ this underscores the benefits of trimerization. As the hydrophilicities (log *D*_7.4_) of both compounds are virtually identical, any differences regarding their behavior in a biological context should therefore be considered a result of altered affinity or molecular size.

**Table 1:** Integrin affinities (expressed as 50 % inhibition concentrations, IC_50_), IC_50_‐based selectivities, and octanol/PBS distribution coefficients (log *D*_7.4_). Affinities were determined by ELISA on immobilized integrins,^31^ using the non‐radioactive ^69/71^Ga^III^ complexes, where applicable.

Compound	IC_50_ (95 % confidence interval) [nm]		Selectivity for α_v_β_6_	log *D*_7.4_
	α_v_β_6_	α_v_β_8_	over α_v_β_8_	
^68^Ga‐TRAP(SDM17)_3_	0.26 (0.21–0.40)	172 (96–312)	662	−4.08±0.07
^68^Ga‐NOTA‐SDM17	7.4±1.1^a^	366±32^a^	49	−3.91±0.07
SDM17	1.3±0.2^a^	174±31^a^	134	n.a.

[a]: IC_50_ data for Ga‐NOTA‐SDM17 and SDM17 were taken from the literature and are shown as reported therein.^12^

### In‐vivo studies

**Figure 2:**
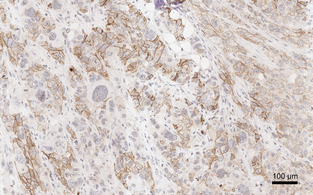
β_6_ Integrin immunohistochemistry (IHC) of H2009 tumor tissue. Note that β_6_ integrin dimerizes only with the ubiquitously expressed α_v_ chain. Availability of β_6_ is thus limiting and indicative for the distribution of the dimer α_v_β_6_,^35^ obviating a separate α_v_ IHC.

In‐vivo characterization of the radiolabeled compounds by PET and biodistribution studies was done using SCID mice bearing subcutaneous xenografts of the human lung adenocarcinoma cell line H2009. Figure 2 shows that the solid tumors grown in mice exhibit only a moderate β_6_ integrin expression density. This however reflects the situation encountered in stroma‐rich tumor entities like pancreatic ductal adenocarcinoma (PDAC), which possess a comparably low fraction of metabolically active tumor cells per tissue volume^10^ and are thus not easily detectable using the standard PET metabolism tracer 2‐[^18^F]fluoro‐2‐deoxy‐d‐glucose (FDG).^34^ Against this background, α_v_β_6_‐targeted PET radiopharmaceuticals are expected to meet a clinical need preferably if they show a high uptake and imaging contrast even for low and moderate target density per tissue volume. Preclinical results obtained for the H2009 xenograft model are thus likely to correspond to the clinical situation.

**Figure 3:**
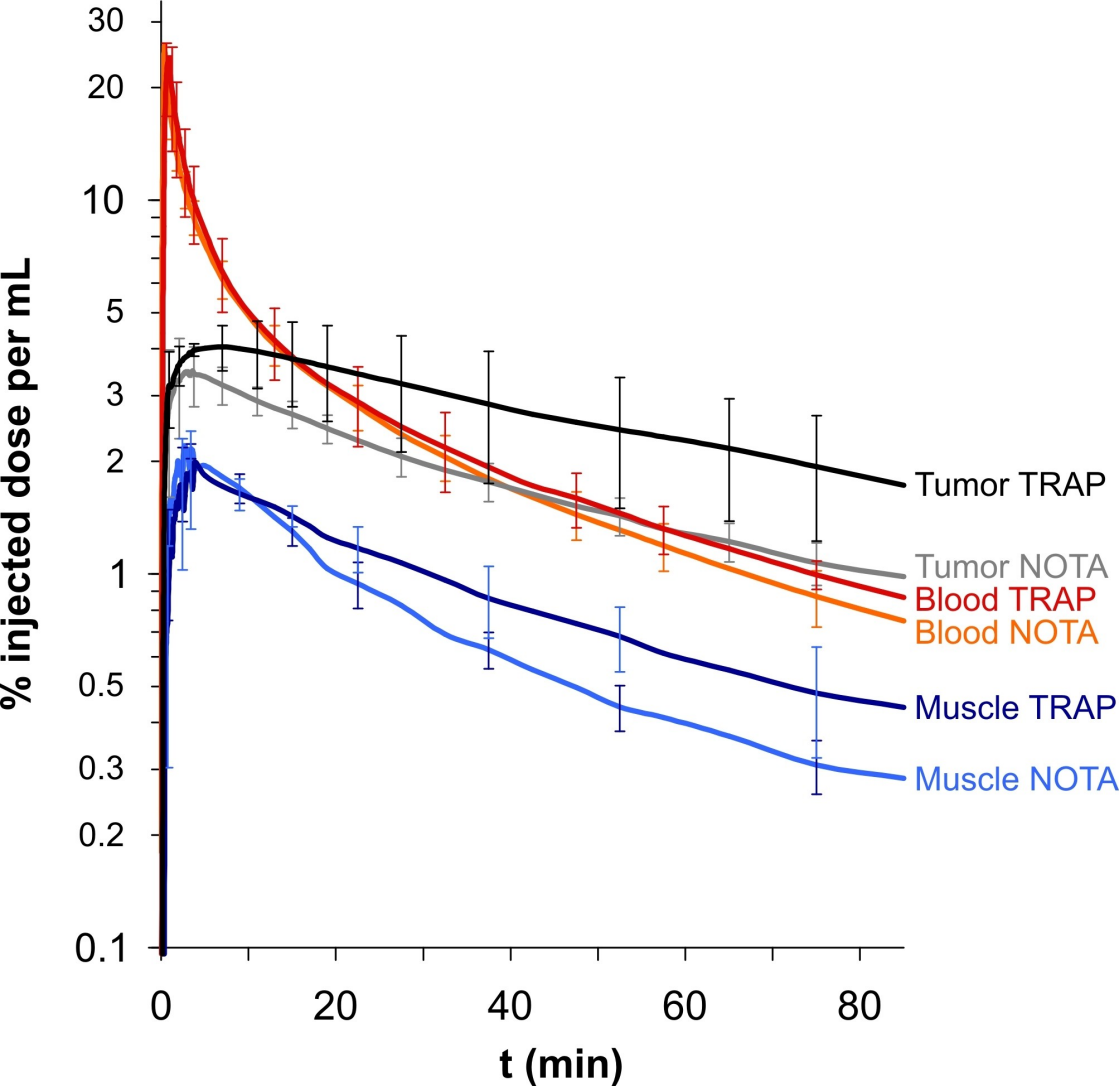
Kinetics of activity distribution in selected compartments for ^68^Ga‐NOTA‐SDM17 (denoted NOTA) and ^68^Ga‐TRAP(SDM17)_3_ (denoted TRAP), derived from dynamic PET data in H2009‐xenografted SCID mice (acquisition time 90 min, *n*=3).

**Figure 4:**
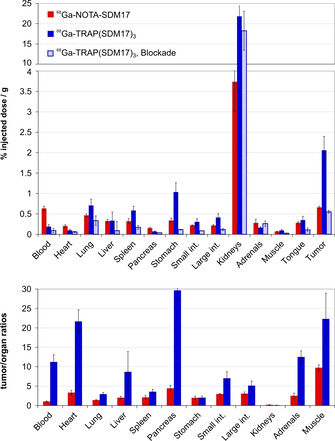
Biodistribution data (90 min p.i.) in H2009 xenografted SCID mice for ^68^Ga‐NOTA‐SDM17 (control: 133±17 pmol, *n*=4) and ^68^Ga‐TRAP(SDM17)_3_ (106±24 pmol, *n*=5; blockade with 50 nmol TRAP(SDM17)_3_ injected 10 min prior to the radiopharmaceutical, *n*=3). Values are given as averages±standard deviation. Data in numerical form are provided in Tables S1 and S2.

Dynamic PET data (Figure 3) illustrate that the trimer ^68^Ga‐TRAP(SDM17)_3_ shows a substantially prolonged tumor retention compared to the monomer ^68^Ga‐NOTA‐SDM17, which is consistent with its higher α_v_β_6_ integrin affinity (Table 1). The trimer is furthermore cleared somewhat slower from muscle, which might be mainly attributed to its larger molecular size, as hydrophilicities (i.e., the log *D*_7.4_) of both compounds are practically similar. At this point, one would also have expected a faster clearance of the smaller monomer from the blood stream, which is typically observed for sets of monomeric/multimeric conjugates of the same targeting vector.^30^, ^36^, ^37^ Instead, biodistribution data shown in Figure 4 confirm a significant blood pool activity for ^68^Ga‐NOTA‐SDM17 after 90 min, which most likely contributes also to the background in other organs and tissues. This however does not indicate a higher non‐specific binding of the radiopharmaceutical but is caused by the aforementioned contamination with non‐complexed ^68^Ga (Figures S4 and S5). Presence of such species in a preparation typically results in an elevated blood pool activity,^38^, ^39^ because free ^68^Ga^III^ is rapidly sequestered by transferrin^40^ and only slowly transported to the liver.

**Figure 5:**
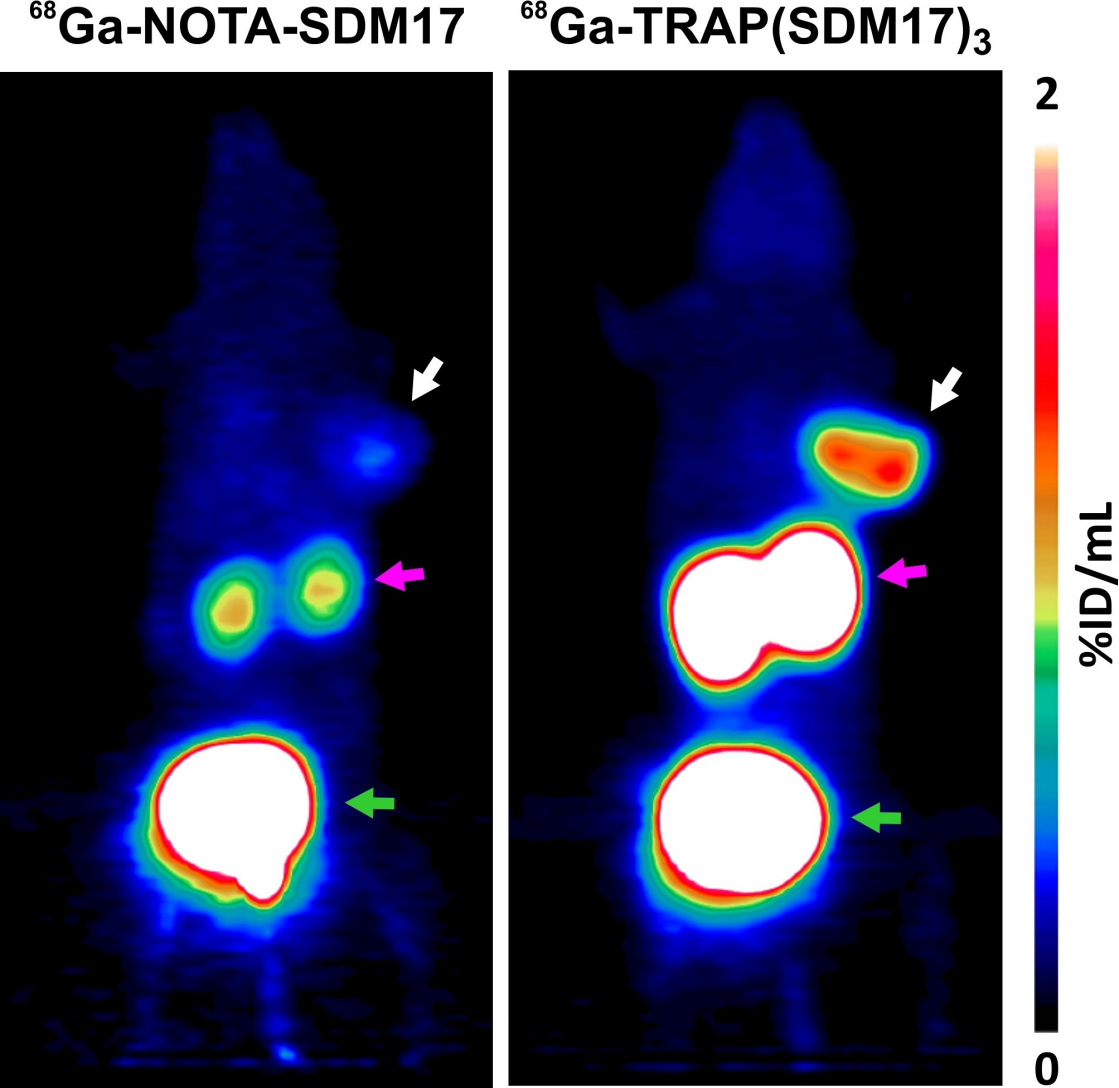
PET images (maximum intensity projections) of the same SCID mouse bearing a subcutaneous H2009 xenograft (human lung adenocarcinoma, positions indicated by white arrow) 75 min after administration of ^68^Ga‐NOTA‐SDM17 (9 MBq, 360 pmol, 24 MBq/nmol) or ^68^Ga‐TRAP(SDM17)_3_ (18 MBq, 270 pmol, 70 MBq/nmol). Purple and green arrows indicate excretion‐related presence of activity in kidneys and urinary bladder, respectively. Scaling, i.e., color coding according to the scale bar, is the same for both images. Recording time 20 min, start 75 min after injection, interval between the scans was 1 day.

Notwithstanding this, the contamination is sufficiently low that it is not producing a visible effect in the PET images. Figure 5 shows that the higher tumor uptake of ^68^Ga‐TRAP(SDM17)_3_ enables a clear visualization of the subcutaneous tumor xenograft, whereas the monomer ^68^Ga‐NOTA‐SDM17 is not capable of delineating the same lesion with satisfying contrast. Of note, the trimer also shows the advantage of much higher tumor‐to‐organ ratios, which is of practical relevance for a possible clinical application. For example, its high tumor‐to‐pancreas and ‐to‐liver ratios (29.7 and 8.7, respectively; Figure 4 and Table S1) appear much more suitable for detection of intrapancreatic or intrahepatic lesions, such as primaries and metastases of pancreatic carcinoma, as compared to the monomer (4.4 and 2.0, respectively; Figure 4 and Table S2).

## Conclusion

^68^Ga‐TRAP(SDM17)_3_ was synthesized by click‐chemistry‐driven trimerization of the α_v_β_6_ integrin‐selective cyclic pentapeptide SDM17 on the TRAP chelator. Compared to the monomer ^68^Ga‐NOTA‐SDM17, the trimer ^68^Ga‐TRAP(SDM17)_3_ showed a higher α_v_β_6_ integrin affinity as well as a higher uptake and longer retention in α_v_β_6_‐positive H2009 tumor xenografts in mice. A high PET image contrast in living subjects (i.e., high tumor‐to‐background ratios) was achieved with ^68^Ga‐TRAP(SDM17)_3_, which encourages the translation into clinics. Notably, several recent reports underscored the potential clinical value of α_v_β_6_‐integrin PET imaging for diagnosis of various carcinomas^41^, ^42^, ^43^, ^44^, ^45^ as well as pulmonary fibrosis^46^ in humans. Hence, we are confident that in view of its favorable preclinical data, ^68^Ga‐TRAP(SDM17)_3_ can contribute to the improvement of healthcare schemes for diagnosis and therapy of carcinomas as well as other conditions associated with α_v_β_6_ integrin dependent dysregulation of the TGF‐β signaling pathway, such as fibrotic diseases.

## Experimental Section

**Materials**: Unless otherwise noted, all commercially available reagents and solvents were of analytical grade and were used without further purification. Protected amino acids were purchased from IRIS Biotech (Germany). Cu(OAc)_2_ **⋅**H_2_O, 4‐pentynoic acid, diisopropylamine (DIPEA) and sodium ascorbate were purchased from Sigma‐Aldrich. 1,4,7‐triazacyclononane‐1,4,7‐triacetic acid (NOTA) was purchased from Chematech (Dijon, France). HATU was obtained from Bachem Holding AG (Bubendorf, Switzerland). HOBt hydrate was obtained from Carbolution (St. Ingbert, Germany). For all radiochemical works, Tracepur® water (Merck) was used. TRAP(azide)_3_ 18 and *o*‐NBS‐l‐Lys(Fmoc)‐OH^47^ were synthesized as described previously.

**Instrumentation**: The synthesis of SDM17‐pentynoic amide was carried out in an ultrasonic bath SONOREX RK 52 H (interior dimensions 150×140×100 mm and operating volume 1.2 L) by BANDELIN electronic (Germany), equipped with timer control for 1–15 min and continuous (∞) operations and built‐in heating control (30–80 °C thermostatically adjustable). Semi‐preparative reversed‐phase HPLC was performed by using a Shimadzu system, consisting of two LC‐20AP quaternary low‐pressure gradient pumps, a SPD‐M30 A photodiode array detector, and a CBM‐20 A system controller. Separations were performed by using a YMC‐Pack ODS‐A, 5 μm, 250×20 mm C_18_ column. Analytical HESI‐HPLC‐MS (heated electrospray ionization mass spectrometry) was performed on a LCQ Fleet (Thermo Scientific) with a connected UltiMate 3000 UHPLC focused (Dionex) on C_18_ columns: S1: Hypersil Gold aQ 175 Å, 3 μm, 150×2.1 mm (for 8 or 20 min measurements); S2: Accucore C18, 80 Å, 2.6 μm, 50×2.1 mm (for 5 min measurements; Thermo Scientific). Linear gradients (5 %–95 % acetonitrile content) with water (0.1 % *v*/*v* formic acid) and acetonitrile (0.1 % *v*/*v* formic acid) were used as eluents. Centrifugation was done with a Heraeus Biofuge 13 benchtop centrifuge. Activities were quantified with a Capintec CRC 15R dose calibrator. Small activities in tissue samples etc. were measured using a PerkinElmer Wizard^2^ 2480 automatic gamma counter. Radio‐TLCs were evaluated using a Bioscan radio‐TLC scanner, consisting of B‐MS‐1000 scanner, B‐EC‐1000 detector with a B‐FC‐3600GM tube.

**Synthesis**: *SDM17‐pentynoic amide*: SDM17 functional monomer was synthesized on solid support by conventional Fmoc/*t*Bu approach, employing an ultrasound‐assisted solid‐phase peptide synthesis (US‐SPPS) protocol.^17^ Rink amide resin (545 mg, 0.3 mmol) was functionalized with *o*‐NBS‐l‐Lys(Fmoc)‐OH (363 mg, 0.6 mmol, 2 equiv) using HBTU (227 mg, 0.6 mmol, 2 equiv) and HOBt (92 mg, 0.6 mmol, 2 equiv) as coupling partners, and DIPEA (209 μL, 1.2 mmol, 4 equiv) as base, in DMF (3.5 mL). The mixture of reactants was added to the resin in a SPPS reactor and then ultrasonicated for 5 min before washing. Fmoc deprotection was carried out by irradiating the resin with ultrasound in the presence of a 20 % piperidine solution in DMF (2×1 min). The linear aminoacidic sequence was elongated by iterative cycles of the aforementioned amide bond coupling reactions and Fmoc deprotection; the completion of each step was qualitatively determined by Kaiser test or TNBS test. After loading the last amino acid, the resin‐bound peptide underwent a Tsuji‐Trost‐mediated allyl ester removal on the glutamic acid side chain. The resin was treated with a solution of tetrakis(triphenylphosphine)palladium(0) (35 mg, 0.03 mmol, 10 % mol) and DMBA (234 mg, 1.5 mmol, 5 equiv) in anhydrous THF (5 mL) for 1 h at rt under argon, and this procedure was repeated once. After being washed with DMF (3×1 min) and dichloromethane (3×1 min), the resin was suspended in a 0.06 M solution of potassium *N*,*N*‐diethyldithiocarbamate in DMF (38 mg in 3 mL of solvent) for 15 min in order to completely remove catalyst traces, and this procedure was repeated twice. At this stage, the α‐amino group of the Arg residue was released, and the cyclization was carried out by adding a solution of PyAOP (469 mg, 0.9 mmol, 3 equiv) and DIPEA (313 μL, 1.8 mmol, 6 equiv) in DMF (5 mL) and allowing the resin to shake for 12 h. Next, the α‐amino group on C‐terminal lysine residue was released by removing the *ortho*‐nitrobenzenesulfonyl (*o*‐NBS) protecting group. This deprotection was performed by adding a clear solution (4 mL) of thiophenol in dry DMF (5 % *v*/*v*) in the presence of 1.5 equiv. (relative to thiophenol) of ultrapure K_2_CO_3_. The obtained suspension was miniaturized by sonication and centrifuged, then the clear supernatant was added to the resin, which was allowed to shake for 10 min. This procedure was repeated a further two times and then the resin was washed exhaustively with DMF (3×1 min), MeOH (3×1 min) and CH_2_Cl_2_ (3×1 min). Final functionalization with the alkynyl‐bearing building block was carried out in a DMF solution (3.5 mL) of pentynoic acid (59 mg, 0.6 mmol, 2 equiv), HBTU (227 mg, 0.6 mmol, 2 equiv) and HOBt (92 mg, 0.6 mmol, 2 equiv) in the presence of DIPEA (209 μL, 1.2 mmol, 4 equiv) and irradiating with ultrasound for 5 min.

The resin was washed with DMF (2×1 min), CH_2_Cl_2_ (2×1 min), and diethyl ether (3×1 min), and the peptide was cleaved from the solid support using a solution of TFA/TIS (95:5, 3 mL) for 3 h at room temperature. The suspension was filtered and the crude product precipitated from the TFA solution by diluting to 35 mL with cold diethyl ether, and then centrifuged (4400 g, 15 min). The supernatant was removed, and the precipitate was suspended again in 35 mL ether as described above. The wet solid was dried for 1 h under vacuum, re‐dissolved in water/acetonitrile (9:1) and purified by RP‐HPLC (solvent A: water +0.1 % TFA; solvent B: acetonitrile +0.1 % TFA; from 10 to 60 % of solvent B over 25 min, flow rate: 10 mL min^−1^). Product‐containing fractions were identified by ESI‐MS, concentrated in vacuo, and lyophilized. The product was characterized by analytical RP‐HPLC (solvent A: water +0.1 % TFA; solvent B: acetonitrile +0.1 % TFA; from 10 to 90 % of solvent B over 20 min, flow rate: 1 mL min^−1^) and HRMS (ESI‐MS) spectrometry. Overall yield: 179 mg (65 %), purity: >95 %, *t*_R_=12.45 min. MW (calcd for C_36_H_57_N_11_O_10_): 803.43. HRMS (ESI‐MS): *m/z*=804.43506 [*M*+H]^+^ (theoretical value: 804.43626; for MS spectra, see Figure S1)

*TRAP(SDM17)_3_*: The trimer was synthesized employing a previously established method.^18^ SDM17‐pentynoic amide (16.1 mg, 20.0 μmol, 3.3 equiv) was added to a solution of TRAP(azide)_3_ (5.0 mg, 6.1 μmol, 1 equiv) and sodium ascorbate (60 mg, 303 μmol, 50 equiv) in a mixture of water and *tert*‐butanol (3:1 by volumes, 400 μL). Copper(II) acetate hydrate (1.45 mg, 7.28 μmol, 1.2 equiv) was added, whereupon a brown precipitate formed immediately. Upon vortexing, the solution turned to a transparent green. The solution was allowed to react for 1 h at 60 °C without stirring. Then, all Cu species were sequestered from the TRAP(SDM17)_3_ compound and the reaction solution by addition of 1,4,7‐triazacyclononane‐1,4,7‐triacetic acid (NOTA) (55 mg, 180 μmol, 30 equiv) dissolved in water (1.5 mL), adjusting to pH 2.2 by using 1 M aq. HCl, and reacted for 1 h at 60 °C. HPLC‐MS was used in all steps for monitoring of reaction progress. TRAP(SDM17)_3_ was obtained as a colorless solid with a yield of 37 % (7.2 mg, 2.2 μmol). RP‐HPLC (gradient: 3–45 % MeCN in water, both containing 0.1 % trifluoroacetic acid, in 20 min, flow rate: 20 mL min^−1^): *t*_R_=16.6 min. MW (calcd for C_135_H_225_N_48_O_39_P_3_): 3235.63. MS (ESI, positive mode): *m/z*=1618.8 [*M*+2H^+^]^2+^, 1080.2 [*M*+3H^+^]^3+^, 810.3 [*M*+4H^+^]^4+^, 648.6 [*M*+5H^+^]^5+^, 540.6 [*M*+6H^+^]^6+^ (theoretical values: 1618.8, 1079.5, 809.9, 648.1, 540.3; for MS spectra, see Figure S2).

**Affinity assays**: The integrin affinities were determined by a solid‐phase binding assay, applying a previously described protocol.^31^ Briefly, flat‐bottom 96‐well enzyme‐linked immunosorbent assay (ELISA) plates (BRAND, Wertheim, Germany) were coated with recombinant human LAP(TGF‐β) in carbonate buffer (15 mM Na_2_CO_3_, 35 mM NaHCO_3_, pH 9.6) at 4 °C overnight. After washing the plates with PBS‐T buffer (phosphate‐buffered saline/Tween20), free binding sites were blocked by incubation with TS‐B buffer (Tris‐saline/BSA). Dilution series of non‐radioactive Ga‐TRAP(SDM17)_3_ (20 μM to 6.4 nM) were prepared and incubated in 1 : 1 mixtures with the respective integrin. Surface‐bound integrin was detected by subsequent incubation with a specific primary antibody and a secondary peroxidase‐labeled antibody (anti‐mouse IgG‐POD, Sigma–Aldrich). After addition of the dye SeramunBlau (Seramun Diagnostic, Heidesee, Germany) and quenching of the reaction by addition of 3 M H_2_SO_4_, the absorbance at *λ*=450 nm was measured with a microplate reader (Tecan Genius, Männedorf, Switzerland). The IC_50_ value for each compound was determined in duplicate and the inhibition curves were analyzed by using OriginPro 9.0 software. The measured IC_50_ values were referenced to the activity of the internal standard RTDLDSLRT:^48^ α_v_β_6_=33 nM, α_v_β_8_=100 nM.

**Radiochemistry**: Fully automated ^68^Ga labeling was done in analogy to a previously described procedure^15^ by using an accordingly programmed robotic system (GallElut+, Scintomics, Fürstenfeldbruck, Germany) which carried out the following steps. A ^68^Ge/^68^Ga‐generator with TiO_2_ matrix (Eckert & Ziegler, Berlin, Germany) was eluted with 0.1 M aq. HCl. A fraction containing the highest activity (1.4 mL, ca. 500 MBq) was collected in a 5 mL conical glass vial, containing 5 or 10 nmol of TRAP(SDM17)_3_ or NOTA‐SDM17, respectively, as well as 50 or 100 μL, respectively, of a solution of 4‐(2‐hydroxyethyl)‐1‐piperazineethanesulfonic acid (HEPES) buffer (2.7 M, prepared from 14.4 g HEPES and 12 mL water), resulting in a labeling pH of approximately 2 or 3, respectively. The vial was heated for 5 min to 100 °C. Purification was done by passing the reaction mixture over a solid phase extraction cartridge (SepPak C8 light), which was purged with water (10 mL). The products were eluted with ethanol (0.5 mL), followed by an ethanol/water mixture (1:1 by volumes, 1 mL). The purity of the radiolabeled compounds was determined by radio‐TLC, using silica impregnated glass fiber chromatography paper (ITLC® by Agilent) as stationary phase, and 0.1 M aq. sodium citrate or a mixture of 1 M aq. ammonium acetate and methanol (1 : 1 by volumes) as mobile phases.

To determine the *n*‐octanol/PBS distribution coefficients (log *D*_7.4_), 650 μL octan‐1‐ol and 650 μL phosphate‐buffered saline (PBS, pH 7.4) were combined in a 1.5 mL Eppendorf tube. Approximately 0.5 MBq of the radiolabeled compound was added and vortexed for 2 min at 2850 rpm using a Vortex Genie2 (Scientific Industries). The samples were centrifuged (11 500 *g*, 10 min), after which 100 μL of the organic phase and 10 μL of the aqueous phase were taken out and the activities of the aliquots were quantified in a γ‐counter. The log *D* values were calculated from the quotients of the measured activities and are given as averages±standard deviation (*n*=10).

**Cell culture**: H2009 human lung adenocarcinoma cells (CRL‐5911; American Type Culture Collection (ATCC), Manassas, VA, USA) were cultivated as recommended by the distributor. Cells were subcultivated after trypsination in a ratio of 1:2–1:5, two to three times weekly in culture medium (DMEM : F12, Biochrom FG4815; 5 % fetal bovine serum, FBS Superior Biochrom S0615; 1 % ITS‐G, ThermoFisher 41400045; 4.5 mM l‐glutamine (final conc.), Biochrom K 0282; 10 nM Hydrocortisone, Sigma H0888, 10 nM β‐estradiol, Sigma E2758; penicillin/streptomycin, Biochrom A 2213).

**In‐vivo studies**: All animal experiments were performed in accordance with general animal welfare regulations in Germany and the institutional guidelines for the care and use of animals. Keeping of the animals, generation of respective tumor xenografts, and ex‐vivo biodistribution studies^49^ as well as μPET imaging^50^ were done following previously described protocols, which are briefly summarized below.

To generate tumor xenografts, 6‐ to 8‐week‐old female CB17 severe combined immunodeficiency (SCID) mice (Charles River, Sulzfeld, Germany) were inoculated with a maximum of 10^7^ H2009 cells (the best results were obtained with 5–7×10^6^) in Matrigel® (CultrexBME, Type 3 PathClear, Trevigen, Gentaur, Aachen, Germany; discontinued in 2019, hence switched to Geltrex™ LDEV‐Free Reduced Growth Factor Basement Membrane Matrix, A1413202, Life Technologies). Mice were used for biodistribution or PET when tumors had grown to a diameter of 8–10 mm (4–5 weeks after inoculation). β_6_ Integrin immunohistochemistry (IHC) was performed as described before.^37^

PET was recorded on a Siemens Inveon small‐animal PET system under isoflurane anesthesia. The animals were injected with between 9 and 18 MBq (200–400 pmol) of the ^68^Ga‐labeled compounds into the tail vein, whereupon PET was either continuously recorded in list mode for 90 min while anesthesia was maintained (dynamic scan, reconstructed as multiple frames), or the animals were allowed to wake up with access to food and water and scanned 75 min p.i. for 20 min with refreshed anesthesia (static scan, reconstructed as single frame). Time between scans shown in Figure 5 was 1 day. Data were reconstructed using Siemens Inveon Research Workspace software, employing a three‐dimensional ordered subset expectation maximum (OSEM3D) algorithm without scatter and attenuation correction. Images of static scans were exported as maximum intensity projections (Figure 5). Time‐activity curves (Figure 3) were obtained by generating isocontour regions of interest (ROI) for the tumor and the heart content (i.e., blood), as well as defining two spherical ROIs (each 23.4 mm^3^) in the thigh area (muscle), followed by plotting of average activity per volume in these ROIs over time.

For biodistribution studies, the mice were administered approximately 120 pmol (3–8 MBq, depending on radiolabeling yield and decay) of the radiopharmaceuticals into the tail vein and allowed to wake up with access to food and water. For blockade, 50 nmol of TRAP(SDM17)_3_ was administered 10 min before tracer injection. Animals were sacrificed 90 min after injection, blood was immediately taken from the heart with a syringe, and the organs of interest (heart, lung, liver, spleen, pancreas, stomach (empty), small intestine (empty), large intestine (empty), kidneys, adrenals, muscle, tongue, tumor, tail) were dissected. The activity in weighed tissue samples was quantified by using a γ‐counter. Injected dose per gram tissue (%ID/g) was calculated from the organ weights and counted activities.

## Conflict of interest

The authors declare no conflict of interest.

## Supporting information

As a service to our authors and readers, this journal provides supporting information supplied by the authors. Such materials are peer reviewed and may be re‐organized for online delivery, but are not copy‐edited or typeset. Technical support issues arising from supporting information (other than missing files) should be addressed to the authors.

Supplementary
