# Supplementary material for: Click‐Chemistry (CuAAC) Trimerization of an αvβ6 Integrin Targeting Ga‐68‐Peptide: Enhanced Contrast for in‐Vivo PET Imaging of Human Lung Adenocarcinoma Xenografts
Source: Chembiochem. 2020 Jun 9;21(19):2836–43. doi: 10.1002/cbic.202000200 (PMC7586803; doi:10.1002/cbic.202000200)
Supplement: Supplementary file 1 — Supplementary [file CBIC-21-2836-s001.pdf]

ChemBioChem

Supporting Information

Click-Chemistry (CuAAC) Trimerization of an $\alpha_v\beta_6$ Integrin Targeting Ga-68-Peptide: Enhanced Contrast for in-Vivo PET Imaging of Human Lung Adenocarcinoma Xenografts

Neil Gerard Quigley[+], Stefano Tomassi[+], Francesco Saverio di Leva, Salvatore Di Maro, Frauke Richter, Katja Steiger, Susanne Kossatz, Luciana Marinelli,* and Johannes Notni*

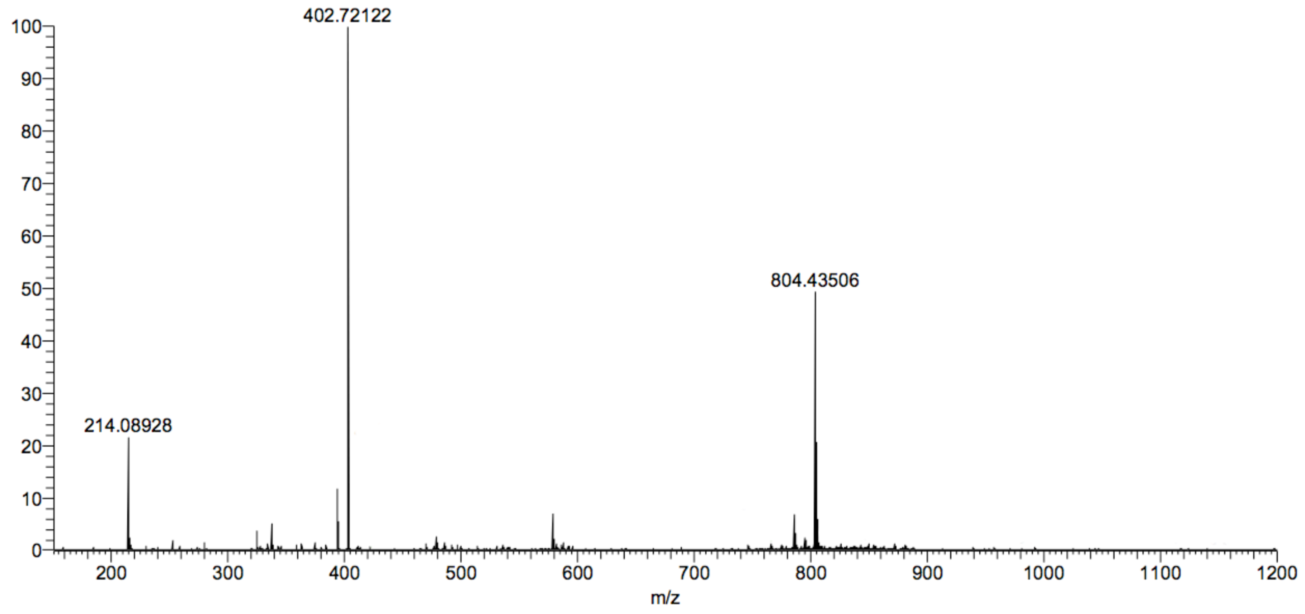

Figure S1: Mass spectrum (ESI, positive mode) for SDM17-pentynoic amide.

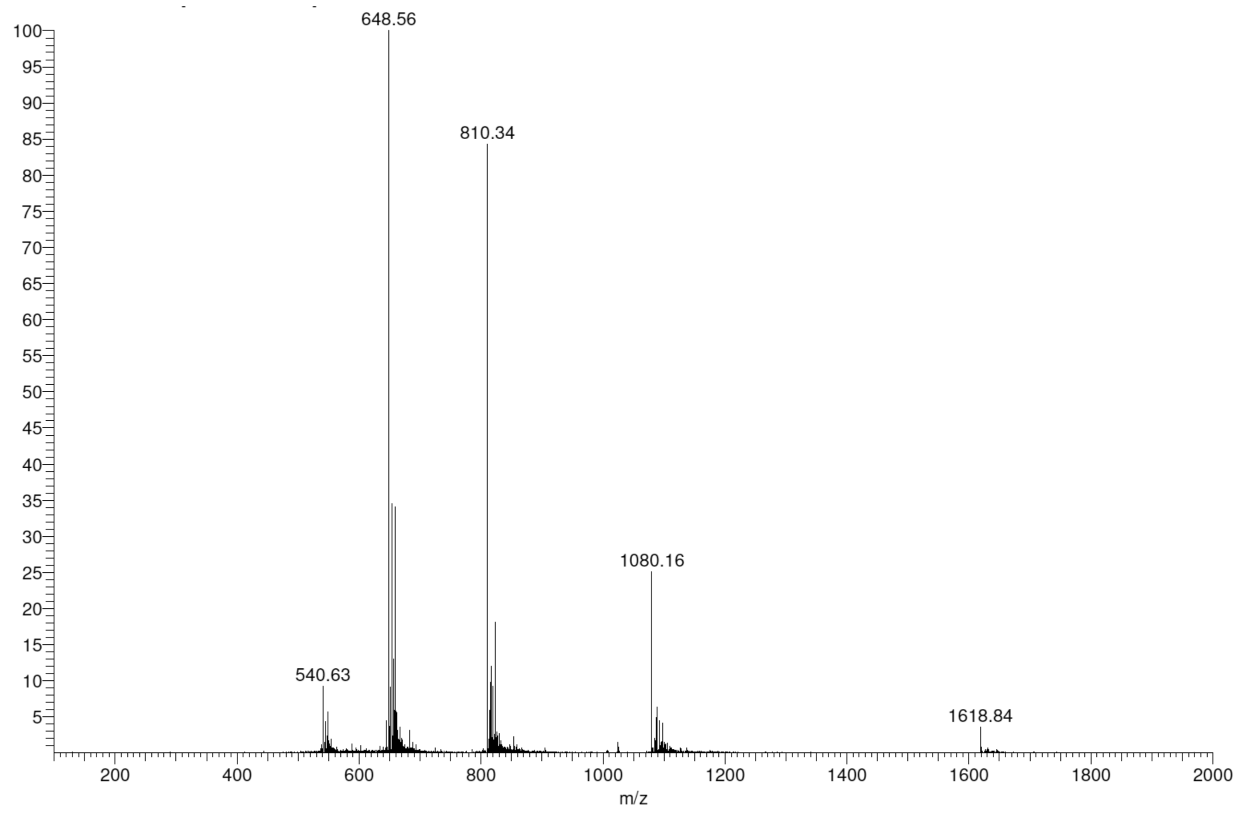

Figure S2: Mass spectrum (ESI, positive mode) for TRAP(SDM17)$_3$.

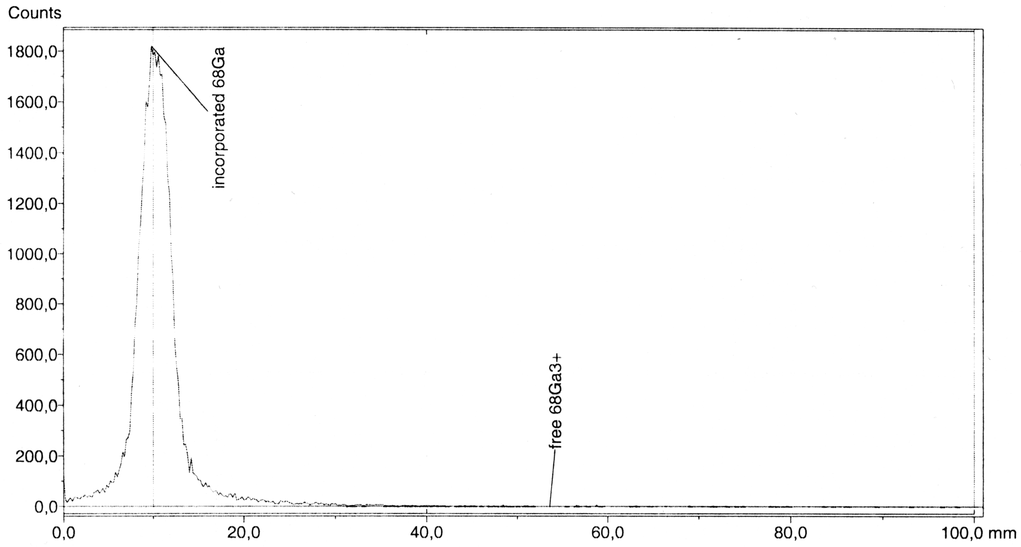

Name	Start (mm)	End (mm)	Retention (RF)	Height (Counts)	Area (Counts)	%ROI (%)	%Total (%)
incorporated 68Ga	0,0	40,0	-0,0	1822,0	41579,0	99,64	99,64
free 68Ga3+	40,0	100,0	0,5	6,0	151,0	0,36	0,36
2 Peaks					41730,0	100,00	100,00

Figure S3: Exemplary radio-TLC for ^{68}Ga-TRAP(SDM17)$_3$ (stationary phase: Agilent ITLC® chromatography paper; mobile phase: 0.1 M aq. sodium citrate, adjusted to pH 5.5).

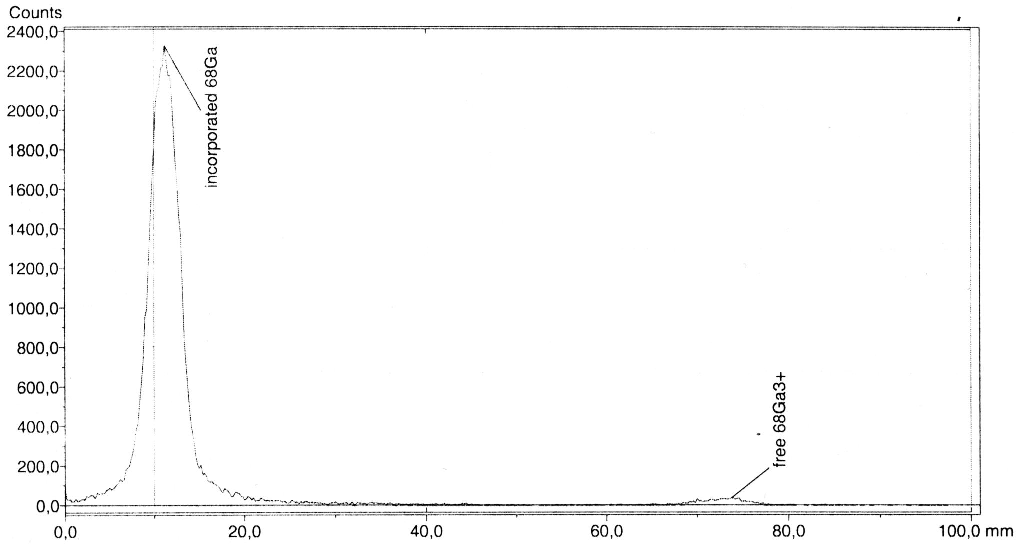

Name	Start (mm)	End (mm)	Retention (RF)	Height (Counts)	Area (Counts)	%ROI (%)	%Total (%)
incorporated 68Ga	0,0	40,0	0,0	2329,0	52531,0	97,51	97,51
free 68Ga3+	40,0	100,0	0,7	37,0	1339,0	2,49	2,49
2 Peaks					53870,0	100,00	100,00

Figure S4: Exemplary radio-TLC for ^{68}Ga-NOTA-SDM17 (stationary phase: Agilent ITLC® chromatography paper; mobile phase: 0.1 M aq. sodium citrate, adjusted to pH 5.5).

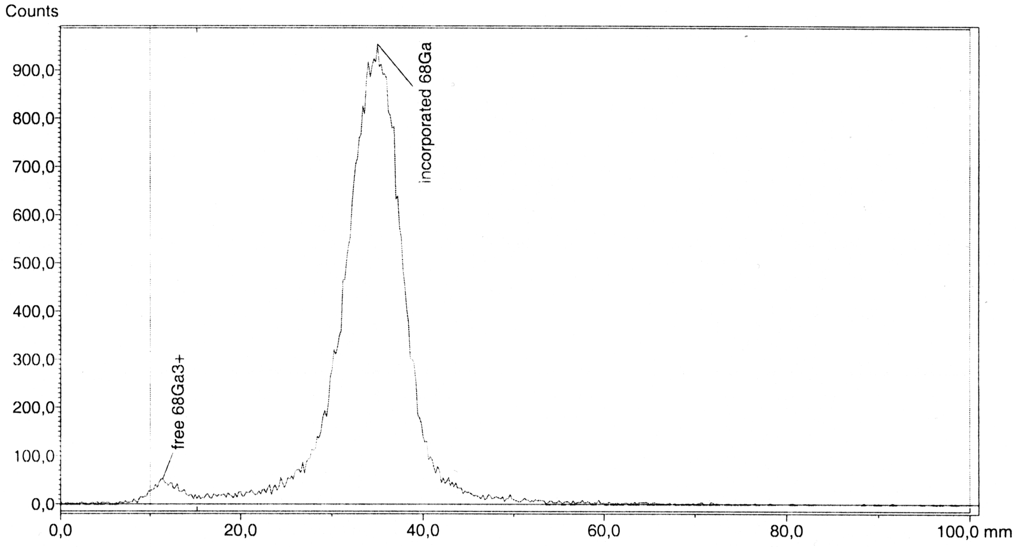

Name	Start (mm)	End (mm)	Retention (RF)	Height (Counts)	Area (Counts)	%ROI (%)	%Total (%)
incorporated 68Ga	0,0	15,2	0,0	53,0	1077,0	2,87	2,87
free 68Ga3+	15,2	100,0	0,3	954,0	36425,0	97,13	97,13
2 Peaks					37502,0	100,00	100,00

Figure S5: Exemplary radio-TLC for ^{68}Ga-NOTA-SDM17 (stationary phase: Agilent ITLC® chromatography paper; mobile phase: 1 M aq. sodium acetate / methanol (1:1 by volumes).

Table S1: Biodistribution data (90 min p.i.) for ^{68}Ga-TRAP(SDM17)$_3$ without (106 ± 24 pmol; n = 5) and with (n = 3) injection of 50 nmol of TRAP(SDM17)$_3$ 10 min prior to tracer administration. Data are given as averages ± standard deviation. %ID/g = percent injected dose per gram tissue.

Organ/Tissue	^{68}Ga-TRAP(SDM17)$_3$		+ 50 nmol cold
	%ID/g	tumor/organ ratio	10 min prior to activity
Blood	0.19 ± 0.05	11.2 ± 1.9	0.09 ± 0.05
Heart (myocard)	0.10 ± 0.03	21.7 ± 3.0	0.06 ± 0.01
Lung	0.71 ± 0.15	3.0 ± 0.4	0.33 ± 0.12
Liver	0.34 ± 0.21	8.7 ± 5.2	0.09 ± 0.22
Spleen	0.58 ± 0.11	3.6 ± 0.6	0.17 ± 0.05
Pancreas	0.07 ± 0.01	29.7 ± 3.0	0.04 ± 0.00
Stomach (empty)	1.04 ± 0.22	2.0 ± 0.4	0.12 ± 0.01
Small intestine (empty)	0.31 ± 0.08	7.0 ± 1.7	0.09 ± 0.00
Large intestine (empty)	0.41 ± 0.09	5.1 ± 1.2	0.12 ± 0.02
Kidneys	21.8 ± 2.7	0.1 ± 0.0	18.2 ± 4.8
Adrenals	0.16 ± 0.02	12.5 ± 1.6	0.26 ± 0.06
Muscle	0.10 ± 0.02	22.3 ± 6.6	0.03 ± 0.01
Tongue	0.35 ± 0.09	6.2 ± 1.8	0.11 ± 0.05
Tumor H2009	2.06 ± 0.34		0.55 ± 0.04

Table S2: Biodistribution data (90 min p.i.) for ^{68}Ga-NOTA-SDM17 (133 ± 17 pmol; n = 4). Data are given as averages ± standard deviation. %ID/g = percent injected dose per gram tissue.

Organ/Tissue	^{68}Ga-NOTA-SDM17	
	%ID/g	tumor/organ ratio
Blood	0.64 ± 0.05	1.0 ± 0.1
Heart (myocard)	0.20 ± 0.03	3.3 ± 0.6
Lung	0.47 ± 0.03	1.4 ± 0.1
Liver	0.33 ± 0.04	2.0 ± 0.3
Spleen	0.32 ± 0.06	2.1 ± 0.4
Pancreas	0.15 ± 0.02	4.4 ± 0.8
Stomach (empty)	0.34 ± 0.06	2.0 ± 0.4
Small intestine (empty)	0.22 ± 0.01	3.0 ± 0.1
Large intestine (empty)	0.22 ± 0.03	3.1 ± 0.4
Kidneys	3.74 ± 0.73	0.2 ± 0.0
Adrenals	0.28 ± 0.09	2.5 ± 0.7
Muscle	0.07 ± 0.01	9.7 ± 0.7
Tongue	0.28 ± 0.03	2.4 ± 0.3
Tumor H2009	0.66 ± 0.03	
